# Impact of tacrolimus peak concentration on kidney graft outcomes

**DOI:** 10.3389/ti.2026.16212

**Published:** 2026-08-17

**Authors:** Quentin Monchal, Guillaume Claisse, Nicolas Maillard, Christophe Mariat

**Affiliations:** 1 Centre Hospitalier Universitaire (CHU) de Saint-Étienne, Saint-Etienne, France; 2 Universite Jean Monnet Saint-Etienne, Saint-Étienne, France

**Keywords:** kidney transplantation, tacrolimus, pharmacokinetics, tacrolimus concentration-to-dose ratio, tacrolimus exposure, graft survival

## Abstract

Low tacrolimus (Tac) trough concentration-to-dose ratio (C0/D ratio) identifies kidney transplant patients with high level of Tac metabolization and is associated with poorer outcomes. We hypothesized that fast metabolization is associated with higher maximal Tac blood concentration (Cmax) and that Tac Cmax has a detrimental effect by itself. We retrospectively selected consecutive kidney transplant patients who (i) were treated by Tac, (ii) had systematic pharmacokinetic evaluation at 3- and 12-month posttransplant, and (iii) with a minimal follow-up of 5 years. Association between Tac Cmax/C0 with traditional transplant outcomes was analysed. 519 patients with a median follow-up of 8 years were included. Fast metabolizers displayed significantly higher median Cmax (20 vs. 17 ng/mL p < 0.001). Death-censored graft survival (DCGS) was significantly lower for patients with the highest Cmax/C0 values (Log rank, p = 0.05). In multivariate cox analysis, higher Cmax/C0 was independently associated with DCGS (HR = 1.37 [1.01; 1.87], p = 0.043). Our data support the hypothesis that exposure to high Cmax is detrimental and that fast metabolizers exhibit higher Tac Cmax even in situations where Tac C0 is not elevated. Tac formulations prone to mitigate pharmacokinetic peak could thus be particularly beneficial to fast metabolizers and should be evaluated in this indication.

## Graphical Abstract

**Figure:**
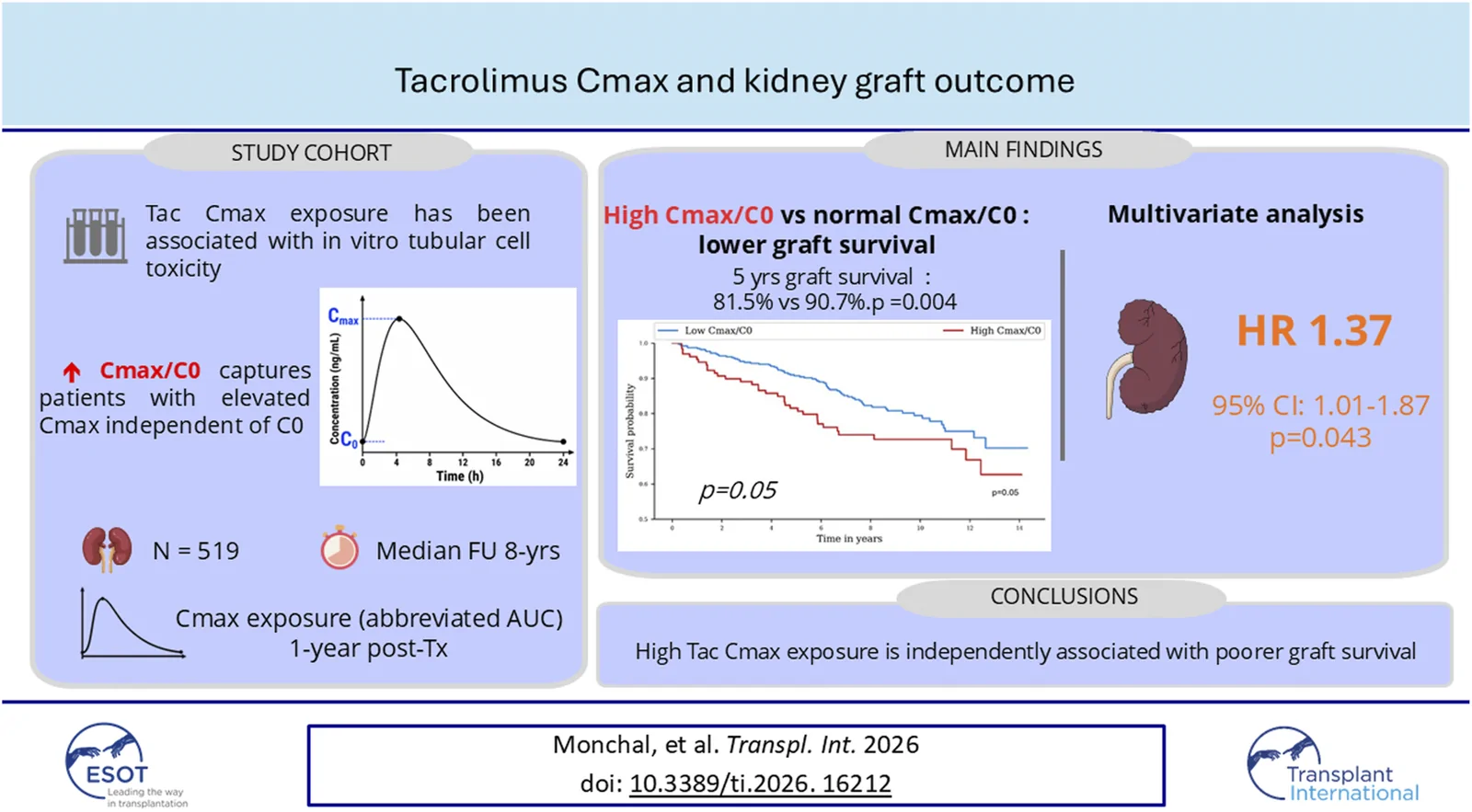

## Background

The calcineurin inhibitor tacrolimus (Tac), when combined with mycophenolate mofetil and glucocorticoids, constitutes the standard immunosuppressive regimen for kidney transplant recipients [1, 2]. Tac is characterised by a narrow therapeutic index: low exposure is associated with higher risk of rejection [3] whereas overexposure leads to acute toxicity [4, 5] as well as to chronic graft injury [6]. In addition, Tac metabolism is subject to substantial intra- and inter-individual variability [7]. In practice, this translates into the necessity to carefully and regularly monitor Tac exposure in transplanted patients. Tac trough level (C0), being well correlated with 24 h-Tac Area Under Curve (AUC), is usually considered as a valid and clinically useful proxy for Tac exposure [8] even though direct evaluation of Tac AUC is recommended in specific situations [9, 10].

With the aim to better capture the clinical impact of Tac metabolization, a novel metric has recently emerged, the ratio between Tac C0 and Tac daily dose, also known as C0/D ratio [11]. Low C0/D ratio (usually below 1.05) identified so- called “fast metabolizer” patients who require higher Tac dose to achieve the desired C0 [11–13]. Retrospectively, the fast metabolizer status has been associated with lower graft function at 2 and 5 years post-transplantation [8], lower graft survival [12], increased risk of rejection [14], higher incidence of BK nephropathy [15] and histological lesions of interstitial fibrosis/tubular atrophy [16].

While a low C0/D ratio is linked to poorer outcome in kidney transplantation, the precise determinants driving this effect remain largely elusive. In this regard, a detrimental role of Tac maximal concentration (Cmax) could be discussed. Fast metabolizers receive higher doses of tacrolimus and might be exposed to higher Cmax levels (for the same C0) than non-fast metabolizers. This was actually reported in patients with the CYP3A5*1 allele, an allele that confers accelerated Tac metabolization [17, 18]. In the same line, Thölking and colleagues have observed higher C2 Tac blood concentrations in fast metabolizer patients, along with higher renal tubular cells toxicity [19].

While those data suggest a detrimental role of Tac Cmax, no study has specifically assessed the link between exposure to elevated Tac Cmax and graft outcome. We thus sought to retrospectively investigate the association between Tac Cmax, C0/D ratio and graft outcome in a cohort of kidney transplant patients who had Tac abbreviated pharmacokinetics as part of their routine follow-up.

## Patients and methods

### Study population

We conducted a retrospective analysis, considering all patients who underwent kidney transplantation between December 2008 and December 2016 at Saint Etienne University Hospital, France, with a minimum follow-up period of 5 years. Outcome data were collected up to December 31, 2021.

This study was formally approved and registered by our local ethics committee “Terre d’Ethique” under reference number IRBN902023/CHUSTE.

Patients under the age of 18 or who had lost their graft within the first 3 months post-transplantation or who received cyclosporine as a maintenance regimen were not included in the analysis. Tac-treated patients were considered eligible for the analysis regardless of the formulation used. Induction therapy was selected based on the immunologic risk profile of the recipient. Maintenance therapy primarily consisted in tacrolimus, mycophenolate mofetil, and steroids. Tacrolimus dosing commenced at 0.1 mg/kg/day, targeting a trough level of 8–12 ng/mL during the initial month, 6–10 ng/mL for the first 3 months, and 4–8 ng/mL thereafter.

Data, collected from medical records, were recipient’s age, gender, weight and height, underlying nephropathy, dialysis status, history of previous kidney transplants, medical history, donor’s age, gender, donor type, expanded criteria donor status, medical history, and morphological data. In addition, type of induction therapy, HLA mismatch in A, B, and DR loci, PRA and pre-existing donor-specific antibodies (DSA), ABO incompatibility, ischemia times, use of machine perfusion, and occurrence of delayed graft function were collected.

### Measurement of tacrolimus serum concentration

In our department, patients routinely undergo tacrolimus pharmacokinetic (PK) evaluation at month 3 and month 12 and every year post-transplant. PK evaluation consists in a 4 time-points measurements: before the morning dose of Tac (C0), and 30, 90 min and 180 min after Tac taking. Tac blood concentration was evaluated using HPLC-MS-MS. Analyses were performed on a Waters Acquity H-Class UPLC system coupled to a Waters Xevo TQD triple quadrupole mass spectrometer (Waters, Saint-Quentin-en-Yvelines, France). The analytical method was fully validated according to the U.S. Food and Drug Administration Bioanalytical Method Validation Guidance for Industry. Furthermore, the tacrolimus assay is accredited by the French Accreditation Committee (COFRAC) in accordance with the ISO 15189 standard for medical laboratories.

For the present analysis, we used the median of M3 and M12 PK values. Cmax was defined by the maximum concentration measured at 90 or 180 min, a time period likely to capture Tac peak concentrations irrespective of its formulations [8].

Two different ratios were considered: the classical C0/D ratio in order to identify fast metabolizers (C0/D < 1.05) and the Cmax/C0 (i.e., Tac peak over trough level) in order to identify patients exposed to elevated peaks despite adequate C0.

### Clinical outcomes

The primary endpoint was death-censored graft survival. Secondary endpoints were patient survival, onset of *de novo* DSA, acute or chronic rejection, histologically proven CNI nephrotoxicity, BK virus associated nephropathy, infectious, cardiovascular, metabolic and carcinologic complications.

### Statistical analysis

All statistical analyses were performed using the R statistical software (R core team 2019) or Easymedstat (version 3.29; www.easymedstat.com). Data were expressed as mean ± SD if normally distributed or median (Q1; Q3) otherwise. Categorical variables were presented as frequency and percentages. Pairwise comparison repeated-measures analyses were performed with Friedman’s test. If the null hypothesis of Friedman’s test was rejected, post-hoc pairwise analyses were performed with Nemenyi’s test (alpha risk was set to 5%). Comparison of baselines characteristics was done using unpaired Student’s t-test, Welch t-test or Mann-Whitney U test for continuous parameters, and chi-squared or Fisher’s exact test for categorical parameters (alpha risk was set to 5% and two-tailed tests were used). Kaplan-Meier method was used to estimate survival probabilities with their pointwise 95% confidence intervals. The Logrank non-parametric test for comparison of survival distributions was used to compare survival differences between groups (alpha risk was set to 5.0%). Cloglog fixed point method was used to compare survival probabilities between groups at different time points. Survival models were built by Cox methodology, to estimate the association between Cmax/C0 with several survival endpoints (patient survival, graft survival, death censored graft survival, survival without acute rejection, chronic rejection…). A multivariate Cox model was built in a forward manner, retaining variables associated with death-censored graft survival at a p < 0.10.

## Results

### Population characteristics

Between December 2008 and December 2016, a total of 559 kidney transplantations were performed at the University Hospital of Saint Etienne, France. Among these patients, 519 met the inclusion criteria and were retained for the analysis. Table 1 displays the characteristics of the study population categorized on the C0/D ratio. Median C0/D ratio was 1.54 (Q1 1.06; Q3 2.16), with 24% of patients classified as fast metabolizers (C0/D < 1.05). Fast metabolizers were notably younger (p = 0.004) and less likely to have received expanded criteria donor (ECD) grafts (p = 0.01). There was no significant difference observed in the immunosuppressive regimen between groups with the vast majority of patients receiving corticosteroids, mycophenolate mofetil, and extended-release tacrolimus formulation (ADVAGRAF^TM^ was the unique extended-release tacrolimus formulation used in this study). Pharmacokinetic profiles did not differ significantly according to tacrolimus formulation, Supplementary Table S1.

**TABLE 1:** Comparison of baseline characteristics and pharmacokinetic parameters according to metabolization status.

Characteristics	Fast metabolizers C0/D ratio <1.05 N = 126	Slow metabolizers C0/D ratio ≥1.05 N = 393	*p* value
Demographics (recipient) Age (years) Gender (male)	52.2 ± 13.7 73 (57.9)	56.1 ± 13.5 262 (66.67)	0.004 0.094
Pre-transplant comorbidities Hypertension Diabetes	95 (75.4) 26 (20.6)	348 (88.6) 6 (15.5)	<0.001 0.23
Donor Age (years)	55.4 ± 14.2	56.8 ± 5.9	0.122
Donor type Living donor Brain dead donor	19 (15.1) 107 (84.9)	40 (10.3) 353 (89.7)	0.188
Expanded criteria donor	43 (34.13)	185 (47.8)	0.01
Immunology PRA >20%	13 (10)	65 (16)	0.16
Induction therapy ATG Anti IL-2 receptor Other	32 (25.4) 94 (74.6) 0	94 (24.0) 294 (75) 4 (1)	0.716
Immunosuppressive drugs (1year)	​	​	​
Corticosteroid Mycophenolate mofetil Tacrolimus IR-tacrolimus ER-tacrolimus	90 (71) 108 (86) 23 (18) 94 (74)	268 (68 351 (89) 83 (21) 288 (73)	0.308 0.538 0.623

### Tacrolimus pharmacokinetics

**FIGURE 1:**
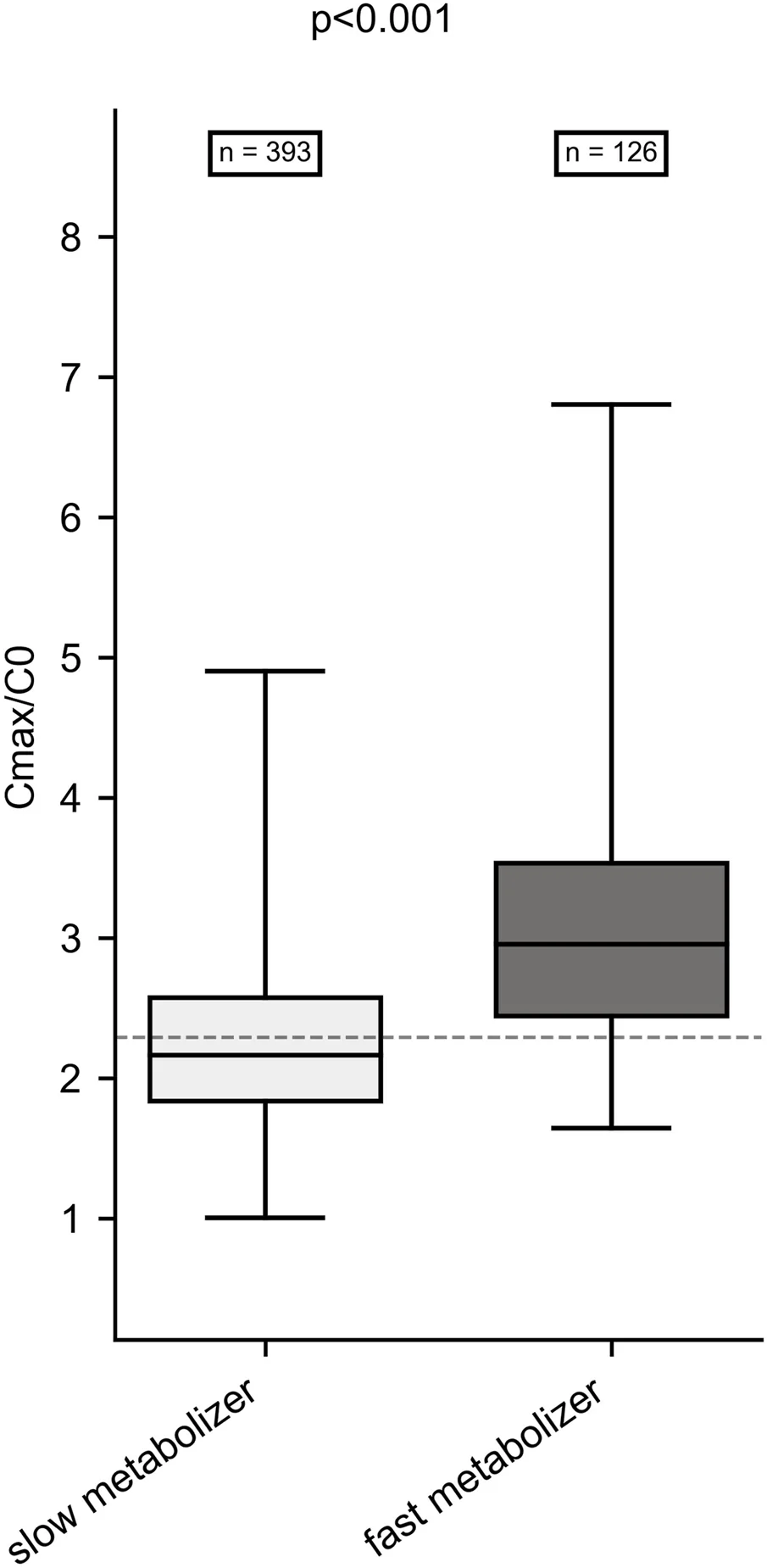
Cmax/C0 ratio according to metabolization status. The boxplot displays the distribution of Cmax/C0 ratio in slow (light grey) and fast (dark grey) metabolizers (C0/D ±1.05).

Table 2 provides a summary of Tac PK parameters. Fast metabolizers exhibited lower C0 [6.8 (Q1 5.8; Q3 7.9) vs. 7.9 (Q1 6.8; Q3 9.2); p < 0.001], while receiving higher doses of Tac [8.5 (Q1 7.5; Q3 10) vs. 4.5 (Q1 3.5; Q3 5.5); p < 0.001], resulting in a lower C0/D ratio (p < 0.001). Interestingly, Cmax was significantly higher in patients classified as fast metabolizers [20.0 (Q1 16.0; Q3 25.4) vs. 17.1 (Q1 13.5; Q3 20.6); p < 0.001]. Tac Cmax/C0 ratio was 2.29 (Q1 1.93; Q3 2.81) in the entire cohort and was notably higher for fast metabolizer patients [2.96 (Q1 2.44; Q3 3.53) vs. 2.16 (Q1 1.84; Q3 2.58); p < 0.001]. We considered the cut-off value of 2.81 (corresponding to the third quartile) to distinguish patients with low (<2.81) and high (>2.81) Cmax/C0 values. Using this cut-off, 56.4% of fast metabolizers exhibited an elevated Cmax/C0 as compared to 15.0% for slow- or normal metabolizer patients (p < 0.001) (Figure 1; Table 2).

**TABLE 2:** Pharmacokinetic parameters.

Variables	Fast metabolizers C0/D ratio <1.05 N = 126	Slow metabolizers C0/D ratio ≥1.05 N = 393	*p* value
C0 (ng/mL)	6.8 (5.8–7.9)	7.9 (6.8–9.2)	<0.001
Dose (mg)	8.5 (7.5–10)	4.5 (3.5–5.5)	<0.001
C0/D	0.84 (0.7–0.95)	1.80 (1.41–2.40)	<0.001
Cmax (ng/mL)	20.0 (16.0–25.4)	17.1 (13.5–20.6)	<0.001
Cmax/C0	2.96 (2.44–3.53)	2.16 (1.84–2.58)	<0.001
Cmax/C0 >2.81	71 (56.4%)	59 (15.01%)	<0.001

### Survival analysis

**FIGURE 2:**
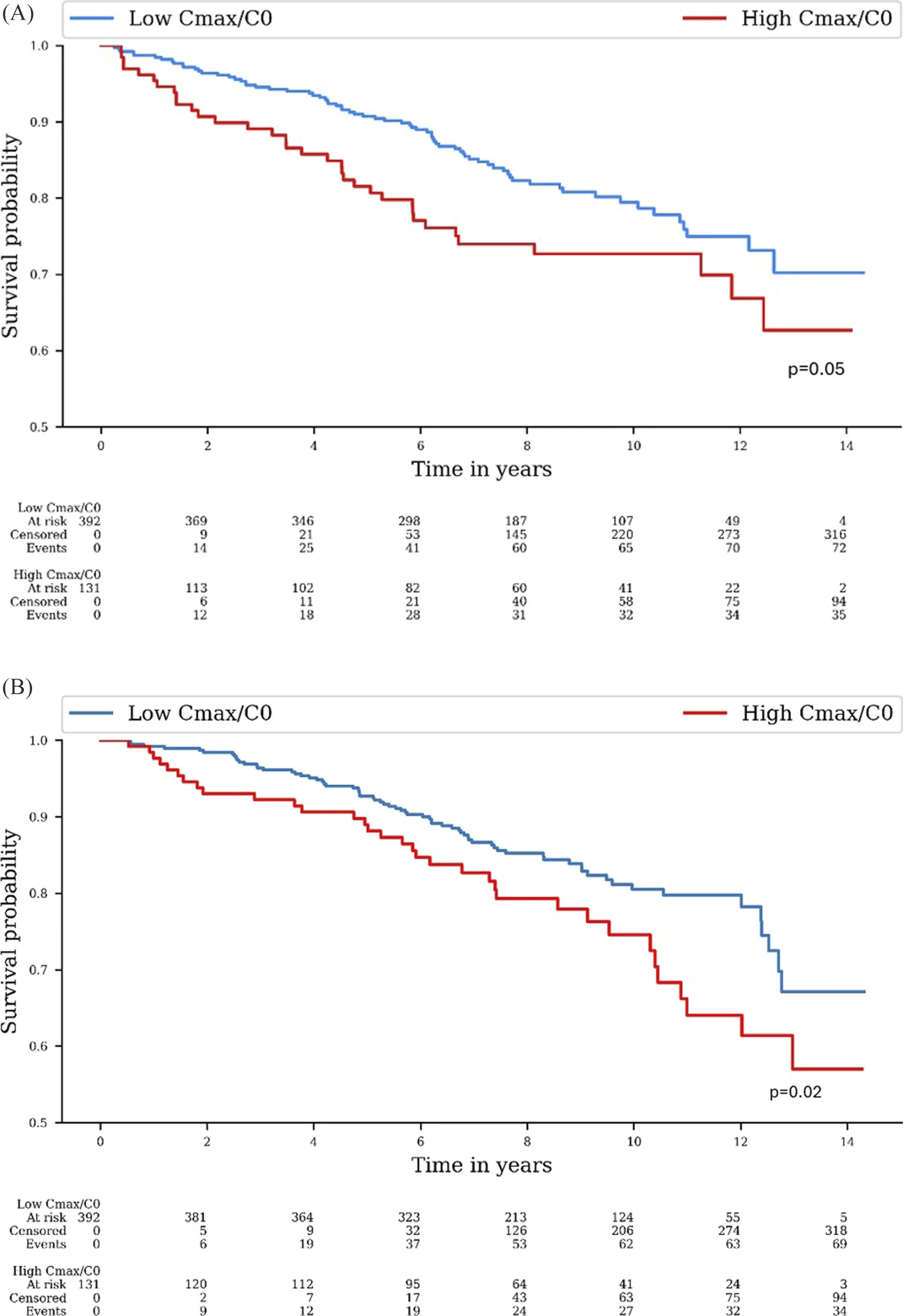
Kaplan-Meier survival curves according to Cmax/C0. Survival analysis between low (<2.81, blue curve) and high (>2.8, red curve) Cmax/C0 for **(A)** death censored graft survival and **(B)** overall survival.

Over the study period, there were a total of 114 deaths and 111 death-censored graft losses. Kaplan-Meier curves for death-censored graft and patient survival according to Cmax/C0 ratio ±2.81 are displayed in Figure 2. Death-censored graft survival (Figure 2A) and patient overall survival (Figure 2B) were significantly lower in patients with a Cmax/C0 ratio >2.81 (p = 0.05 and p = 0.02, respectively). At 5 years, death-censored graft survival was 90.7 (95% CI: 87.9–93.7) in the low Cmax/C0 group compared to 81.5% (95% CI: 75.0–88.7) in the high Cmax/C0 group (p = 0.004). Overall survival was 5.8% lower in the high Cmax/C0 group, with rates of 86.7% (95% CI: 81.0–792.8) versus 92.5% (95% CI: 0.90–0.95), respectively (p = 0.04).

In univariate Cox regression model, higher Cmax/C0 ratio was significantly associated with reduced death-censored graft survival (HR 1.26 [95% CI: 1.004–1.57]; p = 0.046) Table 3. In multivariate analysis, after adjusting for age, diabetes, preemptive status, ECD status and cPRA, the association between Cmax/C0 ratio and lower death-censored graft survival remained significant (HR 1.37 [95% CI: 1.01–1.87]; p = 0.043).

**TABLE 3:** Univariate and multivariate Cox regression model to predict death-censored graft survival. Multivariate model was built in a forward manner, retaining variables associated with death-censored graft survival at a p < 0.10.

Variables	Univariate		Multivariate	
	HR (IC 95%)	*p* value	HR (IC 95%)	*p* value
Age	1.03 (1.014–1.047)	<0.001	1.03 (1.00–1.06)	0.042
Gender	1.105 (0.5–1.62)	0.61	​	​
Body Mass index	1.02 (0.98–1.06)	0.33	​	​
Cold ischemia	1 (0.99–1.02)	0.16	​	​
PRA	1.007 (1–1.014)	0.048	1.00 (1.00–1.01)	0.019
Induction treatment	1.45 (0.99–2.1)	0.053	​	​
Diabetes (recipient)	2.34 (1.54–3.57)	<0.001	2.44 (1.41–4.23)	0.001
Living donation	0.76 (0.38–1.50)	0.43	​	​
Preemptive transplantation	0.44 (0.21–0.94)	0.03	0.93 (0.42–2.08)	0.86
ECD donor	2.6 (1.76–3.83)	<0.001	1.54 (0.86–2.77)	0.14
Cmax/C0	1.26 (1.004–1.57)	0.046	1.37 (1.01–1,87)	0.043

ECD, Expanded Criteria Donor; PRA, Panel Reactive Antibody.

### Association between Cmax/C0 and other clinical outcomes

Table 4 summarizes the association between Cmax/C0 and other clinical outcomes in univariate Cox regression model. Higher Cmax/C0 was associated with a higher risk of BK nephropathy (HR 1.52 [1.05–2.21], p = 0,0284). This relationship was not found with other viral infections. High Cmax/C0 ratio was not associated with the occurrence of acute rejections episodes neither the risk of developing *de novo* DSA formation. No significant relationship was observed between Cmax/C0 ratio and histological evidence of CNI-induced nephrotoxicity.

**TABLE 4:** Association between Cmax/C0 and other clinical outcomes in univariate Cox regression model.

Outcome	HR (IC95%)	P value
Early outcomes		
Acute rejection	1.193 (0.972–1.464)	0.091
BK nephropathy	1.518 (1.045–2.20)	0.029
CMV infection	1.168 (0.877–1.554)	0.288
Other viral infection	1.28 (0.837–1.97)	0.257
Infectious event	1.16 (0.93–1.45)	0.185
Late outcomes		
Graft loss	1.163 (0.976–1.388)	0.092
Death	1.163 (0.944–1.474)	0.146
Death censored graft loss	1.258 (1.004–1.574)	0.046
Chronic rejection	0.932 (0.631–1.378)	0.726
DSA *de novo*	1.044 (0.823–1.324)	0.723
Histological toxicity	1.144 (0.879–1.488)	0.317
Cancer occurrence	0.952 (0.776–1.167)	0.635
NODAT	0.71 (0.5–1.02)	0.067
Cardiovascular event	0.70 (0.570–1.039)	0.088

## Discussion

In this study, we took advantage that Tac PK monitoring is routinely realized in our centre, to evaluate a possible detrimental effect of high Cmax exposure on transplant long term outcome. The main result of our study is that high Cmax exposure is significantly associated to decreased graft survival and that this detrimental effect is at least partially independent of Tac C0 and even of Tac AUC.

In the present study, we have first observed - consistently with previous reports- that fast metabolizers transplant patients exhibit significantly higher Cmax compared to slow/normal metabolizers [19]. It was thus tempting to speculate that elevated Cmax was responsible, at least partially, for the poor transplant outcome observed in recipients with low C0/D ratio, keeping in mind that several experimental and clinical data support a model of Tac Cmax induced nephrotoxicity [19]. However, as others previously reported, we were not able to identify a direct association between Cmax and transplant outcome. We believe that this apparent discrepancy can be explained by the fact that high Cmax actually reflects two different clinical situations. First, a scenario in which Tac overexposure is concomitant to high C0. In this scenario, exposition to high Cmax is obvious and likely to be rapidly corrected by decreasing Tac dosing. Second, a scenario in which elevated Cmax coexists with an apparently “normal” Tac C0 (i.e., within the expected range and thus falsely reassuring). In this scenario, elevated Cmax is *bona fide* not detectable and is likely to remain uncorrected. In consequence, testing the hypothesis of a direct toxicity of Tac Cmax may require to preferentially select the subpopulation of patients with a “high Cmax–normal C0” profile.

By re-expressing Cmax as Cmax/C0 ratio, we intended to better decipher the clinical impact of elevated Cmax, the hypothesis being that higher Cmax/C0 ratio confers higher risk. We did observe a statistically significant and independent association between Cmax/C0 and graft survival. In our cohort, an elevated Cmax/C0 clearly appears to be a marker of poor outcome suggesting that Tac Cmax overexposure could be deleterious (with a positive trend irrespective of the C0/D ratio, Supplementary Figure S3). While patients with high Cmax/C0 did not have more histological lesions related to Tac nephrotoxicity, they were significantly more likely to develop BK-associated nephropathy. The exact mechanisms underlying Cmax toxicity will however need to be thoroughly explored in larger and more appropriately designed studies.

Tac PK varies according to Tac formulation [8, 18, 20] and may impact Cmax/C0 ratio. In our study, we did not find any difference in Cmax alone or Cmax/C0 between different Tac formulations but the large majority of our patients (almost 75%) received conventional extended released-Tac. It is known that patients under the LCP formulation have significantly lower Cmax (−31%) especially when they expressed CYP3A5 fast metabolizer alleles [16]. In a retrospective proof of concept study [21], when patients were switched from intermediate release- to LCP formulation early after transplantation, fast metabolizers patients showed a noticeable and persistent increase of eGFR (+4.15 (mL/min/1,73 m^2^) whereas eGFR in slow metabolizers remained stable. While this was not directly evaluated in the present study, our data suggest that a lower Cmax exposure following the switch to LCP Tac might have played a role in renal graft function improvement. This speculation will need to be formally tested in a prospective and controlled manner, especially given that LCP-formulation has so far not demonstrated any consistent benefit over conventional extended-released formulation of Tac.

Interestingly, Tac Cmax/C0 and Tac AUC were poorly correlated (Supplementary Figure S1). Additionally, we did not observed a significant association between high Tac AUC and graft survival (Supplementary Figure S2). Together, those observations suggest that the detrimental effect of high Cmax/C0 is at least partially independent of Tac AUC.

Beyond the impact of Cmax, accumulation of Tac metabolites are also potential factors that could negatively influence graft outcome, especially in fast metabolizers [22, 23]. The increased Tac dosing which is required in fast metabolizers could result in intra renal metabolites accumulation responsible for cellular damages. This hypothesis is currently being explored in the TIPS study (ClinicalTrials.gov Identifier: NCT04526431).

Cmax value was defined in our study as the higher Tac concentration analysed between 2 predefined time points, namely, 90 and 180 min. Strictly speaking, this does not correspond to the real Cmax which would require a full PK analysis, a procedure hardly implementable in clinical practice. However, considering the PK profile of Tac [8, 24], we can reasonably assume that Cmax values determined from our abbreviated PK were close to the real Cmax. Of note, the pharmacokinetic status of each patient was determined from the median Tac concentrations measured at month 3 and month 12. We acknowledge that this relies on the assumption that the pharmacokinetic status is stable over this time-period, which was actually verified for a majority (81%) but not for all our patients.

Our study has other limitations, the first being its retrospective, monocentric nature. The absence of a systematic protocol biopsies policy in our centre may have also accounted for the low rate of histologically documented calcineurin inhibitor toxicity and our inability to demonstrate the histological impact of Tac overexposure that is usually expected [16]. Additionally, physicians had access to Cmax values which could have influenced subsequent prescriptions in adjusting Tac dosing. Finally, Tac PK results did not include AUC measurements. Tac AUC is considered the most reliable parameter to assess Tac exposure [9].

To the best of our knowledge, our study is the first one to have analysed the relationship between metabolization status and Tac peak concentration in regard with transplant outcome. Of note, all Tac measurements were done in the same laboratory at university hospital of Saint Etienne using a HPLC-MS-MS method, limiting biases due to the dosage technique. Another strength of our study lies in its relatively long median follow-up time of 8 years.

In conclusion, our data suggest that high Tac Cmax exposure is independently associated with poorer graft survival and support the hypothesis that fast metabolizers exhibit higher Tac Cmax even in situations where Tac C0 is not elevated.

Tac formulations prone to mitigate pharmacokinetic peak could thus be particularly beneficial to fast metabolizers kidney transplant patients and should be thoroughly evaluated in this indication.

## Data Availability

The raw data supporting the conclusions of this article will be made available by the authors, without undue reservation.
