# Supplementary material for: Impact of tacrolimus peak concentration on kidney graft outcomes
Source: Transpl Int. 2026 Aug 17;39:16212. doi: 10.3389/ti.2026.16212 (PMC13526684; doi:10.3389/ti.2026.16212)
Supplement: Supplementary file 1 [file Table1.docx]

Supplementary Table 1: Main pharmacokinetic parameters measured one year post-transplant according to Tac formulation

| **Variables** | **IR-Tac**  **N = 102** | **ER-Tac**  **N=372** | ***p* value** |
| --- | --- | --- | --- |
| **C0 (ng/ml)** | 7.3 (5.9-8.5) | 7.2(6.0-8.7) | p=0.703 |
| **Dose (mg)** | 4 (3-6) | 5 (3.5-6) | P=0.023 |
| **C0/D** | 1.7(1.17-2.37) | 1.56 (1.08-2.12) | p=0.089 |
| **Cmax (ng/ml)** | 15.2(12.5-22.6) | 17.0 (13.8-20.8) | p=0.396 |
| **Cmax/C0** | 2.28 (1.78-2.98) | 2.38(1.92-2.80) | p=0.887 |
| **AUC** | 232.7 (182.3-309.6) | 239.3(197-281.4) | p=0.108 |
